# Factors associated with loss-to-follow-up of HIV-positive mothers and their infants enrolled in HIV care clinic: A qualitative study

**DOI:** 10.1186/s12889-020-8373-x

**Published:** 2020-03-06

**Authors:** S. Mpinganjira, T. Tchereni, A. Gunda, V. Mwapasa

**Affiliations:** 1 College of Medicine, University of Malawi, Private Bag 360; Chichiri, Blantyre, 3 Malawi; 2 Clinton Health Access Initiative, Boston, USA

**Keywords:** PRIME, PMTCT, Loss to follow up, Mother-infant pairs, Retention, Option B+

## Abstract

**Background:**

In Malawi, loss to follow-up (LTFU) of HIV-positive pregnant and postpartum women on Option B+ regimen greatly contributes to sub-optimal retention, estimated to be 74% at 12 months postpartum. This threatens Malawi’s efforts to eliminate mother-to-child transmission of HIV. We investigated factors associated with LTFU among Mother-Infant Pairs.

**Methods:**

We conducted a qualitative study, nested within the “Promoting Retention Among Infants and Mothers Effectively (PRIME)” study, a 3-arm cluster randomized trial assessing the effectiveness of strategies for improving retention of mother-infant pairs in HIV care in Salima and Mangochi districts, Malawi. From July to December 2016, we traced and interviewed 19 LTFU women. In addition, we interviewed 30 healthcare workers from health facilities where the LTFU women were receiving care. Recorded interviews were transcribed, translated and then analysed using deductive content analysis.

**Results:**

The following reasons were reported as contributing to LTFU: lack of support from husbands or family members; long distance to health facilities; poverty; community-level stigma; ART side effects; perceived good health after taking ART and adoption of other alternative HIV treatment options.

**Conclusion:**

Our study has found multiple factors at personal, family, community and health system levels, which contribute to poor retention of mother-infant pairs in HIV care.

## Background

In 2010, Malawi set an ambitious goal of achieving virtual elimination of HIV mother-to-child transmission by 2015, which entailed reducing mother to child transmission of HIV to less than 5%. As one strategy of achieving this goal, in 2011, Malawi adopted the Option B+ strategy whereby all HIV-positive pregnant women are initiated and maintained on antiretroviral therapy (ART) regardless of their immune-competence.

By 2016, the Ministry of Health estimated that 92% of HIV-positive pregnant women were initiated on ART [1] but only 74% of those women were retained in HIV care at 12 months postpartum [2]. Studies done elsewhere have documented similar rates of Loss-To-Follow-Up (LTFU) in HIV care [3–9]. LFTU breastfeeding mothers, who therefore are not adhering to ART, are at high risk of transmitting HIV to their children. Hence, this threatens Malawi’s goal to achieve virtual elimination of mother to child transmission of HIV. Unsurprisingly, an estimated 3500 children in Malawi became infected with HIV in 2018, mostly due to mother-to-child transmission [1].

Elimination of mother to child transmission of HIV involves many components, including HIV testing of pregnant women, maternal ART initiation for HIV-positive women, ART prophylaxis and early infant diagnosis for HIV-exposed infants, and lifelong HIV care for HIV-positive mothers and infants. Thus, strong retention mechanisms across this cascade of care and treatment services are a critical lever of success in the elimination of mother-to-child transmission of HIV.

Previous studies have explored different reasons for LTFU of HIV-positive mothers and their exposed children. These include: lack of husband support [10–14]; stigma [10, 12, 14–18]; distance and transport costs [10–12, 17, 19]; poor attitude of health workers [3, 17, 20]; food shortage [3, 21]; inadequate counselling [14, 22, 23]; perceived wellness [24–26]; side effects of ART [21, 23]; gender inequality; lack of privacy at ART clinics; lack of access to care and treatment; and involuntary HIV disclosure [10–12, 21, 22, 27].

However, a few Malawian studies considered perspectives of the LTFU women and health care workers who provided care for these women [9, 10, 12, 13, 20, 22]. Hence, the aim of this study was to describe factors associated with loss to follow up, from the perspective of women who were actual LTFUs and healthcare workers who directly provided care to them. This would help to discover other pertinent reasons for LTFU of HIV-positive mothers and their exposed infants, which are not being addressed by current interventions. In addition, it would help understand the mechanisms through which the known reasons operate. This would inform HIV/AIDS program managers to know what other services to add to the HIV care package so that as many women as possible remain in care, hence reduce mother to child transmission.

## Methods

### Study design and study site

This was a qualitative sub-study nested within the PRIME cluster randomized trial, which was conducted in Mangochi and Salima districts, in Southern and Central Malawi, respectively. The PRIME cluster randomized trial compared the effectiveness of three service delivery models for retaining HIV-positive women and their HIV-exposed infants in HIV care. Details of the PRIME study have been described elsewhere [28, 29]. The qualitative sub-study was conducted in fifteen of the thirty health care facilities that participated in the PRIME trial.

### Study population

This sub-study population comprised HIV-positive women enrolled in the PRIME cluster randomized trial who were documented to have been initiated on ART but had permanently stopped attending HIV care at their designated health facilities any time before 12 months without a documented transfer-out to another health facility. We excluded women who appeared to have dropped out of the HIV care in the study facilities involved in the PRIME study but had actually continued to receive ART elsewhere, based on mother’s report and health profile book. In addition, we included health workers based at the health facilities where the women were receiving HIV care. We excluded health workers who had not been working at the health facilities at the time when the PRIME study was being implemented.

### Sample size and selection of study participants

Our total target sample sizes were 30 LTFU women and 30 health care workers. A multistage sampling method was used to identify women who were LTFU. Firstly, 5 health facilities were randomly selected from each of the three study arms of PRIME cluster randomized trial, making a total of 15 health facilities from the two districts. Thereafter, a list of enrolled women who were LTFU at the selected facility was obtained from the PRIME study registers. A maximum of two LTFU women per facility were randomly selected from this list. In addition, two health care workers, who worked in the maternal and child health clinics where the PRIME cluster randomized trial was conducted, were conveniently selected from each sampled study health facility.

### Data collection

Data collection was conducted from July to December 2016. The data collection team comprised of 10 research assistants, (5 females and 5 males) who had extensive training and experience in conducting qualitative interviews. Firstly, the research assistants checked the health facility records such as ART master cards and registers to verify whether the identified LTFU women had indeed stopped receiving HIV care. Thereafter, they initiated the process of tracing the women, with the assistance of nurses, health surveillance assistants and community volunteers (expert clients). The health surveillance assistants and the community volunteers, then informally approached an eligible mother to assess whether she was willing to participate in the follow-up interviews. If she expressed willingness, arrangements were made for trained research assistants to visit her in her home or at an alternative suitable location as determined by the participant. The interviews were held at any location, either at the health facility or in the community, as the clients deemed that their identity and privacy could be safeguarded. On average, an interview took about 30–45 min. Written consent was sought from the women to conduct and audio-record the interviews. The research assistants also recorded the number of LTFU women who were traced but did not grant consent for interviews.

In addition, the research assistants approached and sought consent from eligible health care workers to conduct in-depth interviews. The health care workers chose the preferred time and place for the interviews that minimized disturbances with their work schedules. Refer to **appendix 2** and **appendix 3** for the interview guides.

### Data management

Tape-recorded data from the in-depth interviews were transcribed in verbatim within 72 h of data collection. Two trained independent research assistants transcribed and then translated the data from local languages (Chichewa or Chiyao) into English. A trained research supervisor read and compared the content of the English transcripts with the original ones. If discrepancies were identified, the supervisor resolved them by listening to the original tapes and discussing the same with the research assistants who transcribed and translated the data.

Electronic data (digital recordings, soft copies of transcripts) were stored in a password-protected computer only accessible to the study team. Personal details were safeguarded; digital recordings securely archived and stored in a protected folder in a secure designated computer, with transcriptions pseudonymised, i.e. participants identified only by a pseudonym. Personal details were omitted from transcripts to ensure confidentiality, while key characteristics of participants that may have arisen in the interview process that made them identifiable were changed, e.g. title, location etc. Under no circumstances were study data released to any third party, without the written approval of the Principal Investigator.

### Data analyses

All the transcripts were uploaded and analysed using computerized qualitative data analysis software, NVivo 10. Data analyses were performed by two researchers, with in-depth knowledge on qualitative analyses. Data analyses were conducted using deductive method of content analysis based on pre-determined categories of factors (personal, family, community and health system). Thereafter, subcategories (subthemes) under the major categories were also developed based on previously known factors. The transcripts were coded according to these categories. Data that only fitted with the developed matrix were analysed. To analyse data that did not fit the developed matrix, inductive principles of open coding and abstraction were used to come up with emergent concepts or sub-themes. The new subcategories categories were added to the much broader categories as required. Thereafter, the research scientists compared their coded transcripts. Inter-reliability checks were done to ensure accurate analysis of the data. The initial interpretations were discussed among the study team for validation and re-validated using the coded transcripts. The final interpretations were supported by quotes from in-depth interviews to illustrate specific findings within each particular theme. We have triangulated the findings from both health worker and client responses. Descriptive statistics was used to summarize the respondent’s socio-demographic data.

### Ethical considerations

The study was approved by College of Medicine Research Ethics Committee and World Health Organisation Ethics Review Committee. We obtained consent from our study participants, and ensured participants’ confidentiality and privacy.

## Results

We successfully interviewed 19 of the 30 LTFU clients and all the targeted 30 health care workers. For the 11 missing clients, 5 did not grant consent and 6 could not be traced. Reasons for the missing clients included: relocation, misidentification and failure to grant consent.

Table 1**Showing demographic characteristics of LTFU women and** summarizes the characteristics of the LFTU women and the health care workers who were interviewed. The median age for the women was 36 years and age range of 21–42 years. The majority were married (73%), had attained primary school education (68%) and survived on subsistence farming (63%). The health care workers had a median age of 37 years and an age range of 25–57 years. The average duration of working at a health facility was 7.4 years. The majority of the health care workers were male (53%), married (80%), and were nurses by profession (33%).

### Reasons for LTFU

Several factors were identified as potential reasons for loss-to-follow up, based on the perspectives of the women and health care workers. They were categorized into personal, family, community and health system factors.

### Personal factors

#### Poor understanding of the indication and purpose of ART

Three clients reported that after taking the drugs, they felt well enough hence they decided to stop taking the drugs, as narrated by one client below:

*“ … I started taking treatment. After I had taken enough of it, I felt that my body became well. So, now I stopped taking the treatment; I am just staying. (Lungwena_Client1).*

Consistent with this finding, health care workers reported that most of the women come for antenatal care as healthy and fit women. As such, many women may be initiated on treatment but they end up as LTFU since they do not feel sick. In addition, health care workers reported that some women felt the ART was only to protect the child. Therefore, once their child tested negative for HIV at 9–12 months, they stopped taking medications despite counselling about the risk of infection during breast feeding, as explained below by one HCW:

*“. … … but most drop out after one year when you tell them that their child seems to be negative then they say ‘What’s the point of going to the hospital?’, even if you insist on counselling them to continue coming to the hospital.” (Malukula_HCW2)*

#### Side effects

Three LTFU women reported that they stopped ART and disengaged from HIV care due to various side effects, which they attributed to ART such as heartburns and vomiting, as narrated below by one woman:

*“At that time, I received [*ART*], but as soon as I took it, I awfully vomited. I would also fall sick so seriously. At some point, I stopped; I was not coming due to the same problem. (Katema_Client1)*

One client was influenced to stop treatment and health facility attendance because of the belief that ART caused insanity, as reported below:

*“ … ...when I was taking the treatment at other times, I was having nightmares that were telling me that I should stop taking the treatment. Because they said that if I continue with the treatment, I will go mad. ‘Stop it’, that’s what they told me” (Malukula_Client3).*

#### Poverty

Four women reported interrupted or completely stopped taking of drugs due to lack of food as consequences of poverty as illustrated in the quote below:

*“ … the problem (*that led to stoppage*) is that I lack food due to lack of money, because for a person to eat anything it’s money … . (Mchoka_Client2)*

Health care workers corroborated this notion by affirming that ART needs to be complimented with good nutrition, as narrated below.

*“ … we tell the women that when they are receiving these drugs they are supposed to eat adequately and you find that other women do not come they say ‘I don’t see the reason for coming to the hospital because even if I take the drugs I cannot use them since I am not eating adequately’ … ” (Mafco_HCW1)*

### Family factors

#### Lack of marriage partner support

Most respondents (eleven) reported that their husbands stopped them from taking ART. Health care workers also corroborated this finding. The lack of support in this case ranged from merely refusing to support them financially to physical violence and divorce as evidenced by the quote below:

*“When I went home and told my husband, my husband did not accept.... I just came* [to the hospital] *and said just give me the drugs I should take them and when I saw that things are becoming difficult* [at home] *I just stopped* [taking ART]. *There were quarrels … … and I was being troubled and lacked peace... my husband was shouting at me as to why I went to the hospital and got tested … . all I faced was ill-treatment …… ” (Nankumba_Client3).*

In one instance, the husband stopped the wife from continuing medication because of the belief that ART causes insanity as shown below:

*“ … ‘these things [*ART*] are making people be insane here in Malawi. Moreover, our heads are not functioning properly. They are made from Indian hemp so worthy throwing into the pit latrine’. Then he picked all of them and threw into pit latrine. So, I also just stayed”. (Maganga_Client3)*

In another instance, a woman reported that she stopped taking treatment and continuing health facility attendance due to her husband’s lack of adherence to pieces of advice provided at the health facility.

*“ … ...I felt like I was wasting the drugs. Because I took drugs and he slept* [has sex] *with me every day without using a condom. I felt like I was wasting the drugs since the instructions from the doctors were not being followed … ” (Mchoka_Client5)*

#### Stigma from family members

Both health care workers and LTFU women (four) reported stigma from relatives as one of the factors that led to LTFU. Some relatives failed to provide physical, moral, and psychological support; others were on the forefront spreading the HIV status to third parties. Fearing stigmatization some women opted not to tell their family members about their HIV status, as stated by one of the women below:

*“Yes, because my relatives do not keep secrets that’s why I just told my one sister who I registered here as my next of kin … it’s only two of us; my sister and I who know... But other relatives like my parents; my brothers … don’t know it” (Katema_Client1)*

### Community factors

#### Stigma from community

The problem of stigma extended from the family to the community level. HIV-positive mothers and their children are spoken ill of and are seen as laughing stocks by other members of the community. To avoid stigmatization, other women opted to attend distant ART clinics, some lied about their destination as shown below:

*“When they ask me ‘where are you going?’ I just say ‘aaa I am going just up there, I have a relative at Nankumba and I am going to chat there’. I lie to others that I am going for some other things … ” (Nankumba_Client1).*

Health care workers corroborated these findings, as shown in the quote below:

*“The community lacks proper counselling, once they hear that the woman is on drugs they start gossiping, speaking ill, laughing at her and making jokes about her and her child. So, this affects them and makes them to stop going to the hospital to get drugs” (Malukula_HCW2)*

Apart from stigmatization from the general communities, two LTFU women reported to be discriminated by community leaders as reported by one woman below:

*“.. Some of the problems are that when things come (*relief items*), they do not include us so that we benefit. We are discriminated people … .” (Mchoka_Client2****)***

Health care workers corroborated these findings, as narrated below:

*“Yes, some activities like piece works, public works programmes, they say: ‘Don’t employ the infected, leave them, they can’t work’. The villagers are a big setback. They contribute towards stigma against them” (Katema_HCW1)*

### Alternative treatment options

Two LTFU reported to have sought alternative care from religious and traditional healers and stopped receiving treatment at the HIV clinic, as narrated below.

*“ … I stopped for several months because I have spirits which make me do work of prayers. So, when I take treatment, I don’t find peace but only Bactrim brings me peace.” (Maganga_Client2)*

In addition, health care workers reported instances where women opted to go for traditional healers and stop treatment as shown by the instance below:

*“ … .. Two if not three months ago there was a person who told people to stop taking ARV drugs. Instead, they should be taking his drugs he brought from South Africa. Each patient was advised to drink up to five litres of his drug” (Life_line_HCW2)*

### Health system factors

#### Distance to health facilities

Seven women reported long distance to health facilities as a contributing factor to LTFU. Most of the women had to walk on foot with babies on their back for many hours to reach health facilities. Other women needed to hire bicycle taxis that charge fare ranging from MK500 to MK2000 (approximately US$0.66 to US$2.65) one-way. As narrated below, a woman stopped attending HIV clinics because she could not afford transport to attend clinics.

*“I stopped because of transport … .as I explained where I am staying is not my real home, so here they receive treatment on Friday … … so it’s hard for me to leave every month to come here to receive treatment … .” (Malukula_Client2)*

Health care workers echoed same problem highlighting how most of the health centres serve large catchment areas. Hence, long distance combined with lack of support from husbands and transport expenses lead to women dropping out of care.

*“ … .and others stop because of long distance some have to travel 27 kilometres just to come and collect medicine … .” (Katema_HCW2)*

### Poor attitude of the health care workers

Two women reported that health care workers shouted at them after missing appointments due to other reasons. Hence, they decided to stop attending clinics, as shown below:

*“ … he* [health worker] *started shouting at me that I have stopped coming here up to the extent of saying he will send me home because of this issue … … ” (Phirilongwe_Client1)*

Some health care workers admitted to having shown poor attitude to clients, manifested by shouting and providing poor instructions to clients. The health care workers attributed this poor attitude to high workload:

*“Sometimes even our attitude can affect our clients as well. If you ask some of our clients, they might tell you that ‘I can’t go there because the health workers have a bad attitude’ but all in all we let them know that we are here to help them …*

*” (Monkeybay_HCW2)*

Other infrequently reported reasons by the LTFU women were forgetting clinic appointments and long waiting times at facilities. The health care workers infrequently reported relocation of the women and their families and intermittent shortage of staff at the health facilities.

The following factors were not reported as causes of LTFU: shortage of drugs as well as infant testing supplies and inadvertent disclosure of HIV status by health workers.

## Discussion

Loss to follow-up of HIV-positive women and their children is a major problem threatening the global target of eliminating HIV mother-to-child transmission. This study was nested within a larger study assessing the effectiveness of integrating mother infant pair clinics and Short Message Service-based tracing of defaulting mothers in improving the retention of mother-infant pairs in HIV care [28, 29]. Despite implementation of these interventions, a significant proportion of mothers and their infants were lost to follow up. Our qualitative study found that several factors operating at personal, family, community and healthy system factors contribute to LTFU.

Among the personal factors, non-supportive or abusive spousal behaviour was frequently reported as a crucial factor associated with LTFUs, consistent with findings from other studies [10–14, 30]. Men are considered to be the primary decision makers regarding family matters, not only in Malawi but also many parts of Sub-Saharan Africa [11]. In addition, economic dependence on men gives men power advantage. Our study has shown negative impact of power imbalance and gender inequality on elimination of mother-to-child transmission of HIV programs. Hence, it underscores the need to empower women in health care decision-making and protect them against domestic abuse. In addition, there is a need to come up with appropriate implementation strategies for male involvement that are feasible for the Malawian setting [14, 23, 31–36].

Perceived good health of the clients was another frequently reported factor associated with LTFUs. One study found that women initiated on ART under Option B+ approach were 5 times more likely to drop out of care than those that started ART for their own health [20]. In our study, similar to a previous study, health care workers reported that most women dropped out of care when their infants tested HIV negative during the postpartum follow-up period [26]. This highlights the need for improved counselling of HIV-positive mothers at ART initiation and during follow-up on the long term benefits of ART on the mother’s health and prevention of HIV-mother-to-child transmission [14, 18, 22]. In addition, women also need reminder messages about ART benefits and risks, through appropriate community and mass media channels.

Some of the clients reported various side effects of drugs. Unsurprisingly, actual and perceived side-effects of ART were reported as contributing factors to LTFU, consistent with findings from previous studies [17, 21, 37]. In this study, some clients appeared to be concerned with ART-induced insanity, which, although reported by only two individuals, could potentially have widespread negative consequences at community level. Interestingly, the ART regimen used at the time of the study contained Efavirenz, an antiretroviral drug which is associated with neuropsychiatric disturbance [38–42]. Whether this is just a notion or an actual side effect, is beyond the scope of this study and needs further exploration. Nevertheless, to minimize the impact of ART-associated side effects on LTFU, there is a need to comprehensively counsel clients during ART initiation on importance of ART, expected side effects and their management. Recently (January 2019), Malawi introduced a new ART regimen, which does not contain Efavirenz (Lamuvidine+Tenofovir+Dolutegravir) and expected to be more tolerable than the previous regimen (Lamuvidine+Tenofovir+Efavirenz). The roll out of this regimen may improve adherence and retention in HIV care. Nevertheless, health care workers need to continuously encourage clients to report ART side effects and assist them in identifying appropriate solutions.

At family and community-level, stigma and fear of discrimination were frequently reported as factors associated with LTFUs, consistent with previous findings [10, 12, 14–18, 37].This highlights the need to intensify interventions to reduce HIV-related stigma and discrimination in all domestic and social circles. In 2018, Malawi enacted the HIV Prevention and Management Act (2018), which includes a provision, which penalizes all acts of HIV-related stigma and discrimination. Optimal implementation of this law may have a positive impact on health care provision of HIV positive mothers, and consequently reduce LTFU.

Household poverty characterized by food shortage was reported as an important factor associated with poor ART adherence and LTFU. This was consistent with previous findings in Malawi [21] and Mozambique [3]. This obviates the need for targeting social safety nets to HIV-affected families as a short-term solution and strengthening of economic empowerment programs as a long-term strategy. Recently, Malawi introduced a social cash-transfer program targeting vulnerable households, including those affected by HIV. Nevertheless, roll out of this program has been slow due to resource constraints [43]. The roll out and impact of this program needs to be monitored to examine whether it is having positive impact on ART adherence and retention in HIV care.

Long distance to the health facilities was frequently reported as a contributing factor to LTFU consistent with previous studies [10–12, 17, 19, 37]. This calls for implementation and intensification of differentiated service delivery models [44]. These include community ART groups, fixed community mobile ART clinics, home delivery of ART and multi-month ART prescription among others which can be implemented to compliment the current approach of facility-based model. These interventions are less costly to the clients and reduce number of clinic visits which is favourable to the patients [45–50]. In addition, they likely reduce workload of health care workers hence reduce the probability of speaking unfavourably to clients due to fatigue.

Interestingly, both health care workers and the clients reported that clients disengaged from HIV care and sought alternative health care, which is consistent with findings from another study [21]. The decision to seek alternative care appeared to be influenced by religious teachings and traditional beliefs. This finding suggests the need for continued community sensitization about HIV and its treatment and engagement with key community stakeholders in health care, including traditional and religious leaders. Implementation of the HIV Prevention and Management Act in Malawi which includes provisions for penalizing dissemination of false information on HIV cure, may facilitate stakeholder collaboration in promoting use of evidence-based HIV treatment intervention and ultimately improve ART adherence and retention in HIV care [51].

Weaknesses in HIV service delivery characterized by poor staff attitude was cited as an important factor associated with LFTU. This was consistent with findings from previous studies [3, 15, 17, 20, 37, 52]. Most health care workers attributed the poor attitude to staff shortages and high workload. It is expected that the workload may increase in view of the increasing numbers of HIV + individuals initiated in HIV care due to the newly adopted” Test ad Treat” approach. This calls for optimal and judicious implementation of strategies to reduce workload including task-shifting and differentiated service delivery models [45–50] which explores different methods of ART service delivery. Interestingly, none of the study participants reported shortage of HIV commodities and supplies as an area of concern suggesting the need to focus on the human element of service provision.

In our study, shortage of health commodities; and inadvertent disclosure of HIV status by health care workers were not reported as causes of LTFU despite being reported in other studies [12–14, 22, 23]. This is good news as resource allocation and ensuring constant supply of HIV supplies is backbone in diagnosis and control of spread of HIV through viral suppression. In addition, it indicates the user-friendliness of the HIV service at the facility, which could be facilitating factor at the health system level.

Other infrequently mentioned reasons for LTFU by the health care workers were forgetfulness [19, 21], client mobility [13], waiting times [3, 14] and intermittent shortage of staff [14].

### Strength and weaknesses of our study

A key strength of our study design was the triangulation of perspectives from both LTFU women and their previous health care workers. In addition, it has helped to explain some of the operational mechanisms of some of the already described reasons for LTFU. Main weakness of our study were our inability to interview 37% of the targeted LTFU women. Nevertheless, data saturation was achieved a sample size of 19 and interviewing additional women would have unlikely generated new information. Another shortcoming of the study was the inability to explore perspectives of husbands and relatives and validate the findings from the LTFU women and health care workers.

## Conclusion

Our study has shown factors operating at different levels influencing LTFU. Personal factors include perceived good health, poverty, drug side effects; family factors include lack of husband support, stigma from relatives; community factors include stigma from community, competing treatment alternatives; health system factors include distance to health facility, poor attitude of health care workers.

### Recommendation and policy implication

This study has shown that multiple factors beyond health system factors contribute to loss-to-follow up in resource-limited countries like Malawi. This suggests that multi-faceted and not singular interventions targeting a number of factors are needed to comprehensively address LTFU. These may include couple counselling at ART initiation, community sensitization about HIV to reduce stigma, dispelling ART misconceptions, improving access to and quality of HIV care and targeted social support for HIV-positive women are key interventions for consideration. Nevertheless, optimal combination of these interventions needs to be evaluated through implementation research.

### Supplementary information

**Additional file 1.** Patient In-Depth Interview Guide.

**Additional file 2.** HCW In-Depth Interview Guide.

## Data Availability

The data sets used and/or analysed during the current study are available from the corresponding author on reasonable request.

## Appendix

**Table 1:** Showing demographic characteristics of LTFU women and health care workers

LTFU Women		Health Care Workers	
Variable	Total enrolled *N* = 19)	Variable	Total Enrolled *N* = 30
Age in years		Age in years	
• Range	21–42	• Range	25–57
• Median	36	• Median	37
Education		Gender	
• Secondary	10% (*n* = 2)	• Male	53% (*n* = 16)
• Primary	68% (*n* = 13)	• Female	47% (*n* = 14)
• None	21% (*n* = 4)		
Source of income		Working period in years	
• Farming	63% (*n* = 12)	• Range	1–29
• Business	21% (*n* = 4)	• Mean	7.4
• Nothing	5% (*n* = 1)		
Religion		Cadre	
• Islam	58% (*n* = 11)	• Counsellors	3% (*n* = 1)
• Christians	42% (*n* = 8)	• Clerks	20% (*n* = 6)
		• Health Surveillance Assistants	23% (*n* = 7)
		• Nurses	33% (n=11)
		• Medical Assistants	13% (n=4)
		• Clinical Officers	3% (n=1)

## Abbreviations

ART: Anti-Retroviral Therapy
HIV: Human Immuno-deficiency Virus
LTFU: Loss To Follow Up
PMTCT: Prevention of Mother To Child Transmission
PRIME: Promoting Retention among Infants and Mothers Effectively
