# Supplementary material for: Factors associated with loss-to-follow-up of HIV-positive mothers and their infants enrolled in HIV care clinic: A qualitative study
Source: BMC Public Health. 2020 Mar 6;20:298. doi: 10.1186/s12889-020-8373-x (PMC7060526; doi:10.1186/s12889-020-8373-x)
Supplement: Supplementary file 1 — Additional file 1. Patient In-Depth Interview Guide. [file 12889_2020_8373_MOESM1_ESM.docx]

# Additional file 1 Patient In-Depth Interview Guide

**Instructions for Interviewer**

Your job as an interviewer is to facilitate honest and detailed responses about what the interviewee actually believes about any particular response to the questions below. This is not an exam for participants agreeing to be interviewed. There are no “right” or “wrong” answers. It is permissible to ask a participant to clarify a response if you do not understand. However, do not seek unnecessary clarifications, causing the nature of the original response to change substantively. While the discussion should feel natural, avoid providing too much of your own personal insight, which may lead or sway a participant to reach your own pre-determined conclusion. Your job is to motivate the participant to expand on their own ideas, and allow them to reach conclusions on their own.

The questions below have been designed as to not solicit simple “yes” and “no” answers, but are open-ended in such a way that participants can answer them as they see fit, given their own experience and knowledge of the question. Your role as a guide is to keep participants from straying off topic. To accomplish this, you may ask for details, stories, anecdotes, descriptions of setting, opinions, attitudes, and perceptions about responses to answers that are already on topic. Avoid repeating a question which, you feel, has already been adequately addressed. Thus, **it is not necessary to ask each and every question in the IDI guide in the sequence that has been provided.**

**Introduction for Participant**

You ………………………. (the participant) have been identified as a key stakeholder who can provide information and perspectives on mother-infant pair clinics, and maternal and pediatric HIV care at ………………………. (name of facility from which she dropped-out) as well as other factors that influence compliance with treatment. Please help us by answering the following questions based on your experiences and opinions from the time you were going to the health facility for PMTCT or HIV care.

The questions are intended to help us identify reasons why some mothers and infants disengage or drop-out from PMTCT or HIV care. The discussion will focus on the following factors:

- Personal or family factor
- Community Factors
- Health System factors

Please indicate whether you are willing to answer the questions below, as well as the extent to which you require your participation in this process to be confidential, by answering the following questions.

Do you understand that no identifying information about you will be shared in our final report?

Yes  No

Do you understand that you can choose not to answer any questions that you feel uncomfortable to answer?

Yes  No

1. **Demographic Data**

Date of birth :____/____/_____ (DD/MM/YYYY)

- *If not known*, please provide estimated age :   years

Marital Status : ___________

- Has the woman’s marital status changed over the past 2 years? Yes No
- *If yes*, please specify:________________

Highest Educational Attainment : ___________

Current Occupation : ___________

- Has the woman’s occupation changed over the past 2 years? Yes No
- *If yes*, please specify

Religion : ___________

- Has the woman’s religion changed over the past 2 years? Yes No
- *If yes*, please specify: _________________________________

For how long have you been living in this community?:  days/weeks/months/years *(tick applicable)*

1. **Personal HIV history**

- Tell me about your family situation at home

Probe: Who stays with you?

Probe: How many children do you have?

Probe: How old is your youngest child?

- Have you ever been tested for HIV? Describe how you felt when you had your most recent HIV test

Probe: when was the latest test done? *was it before, during or after pregnancy?*

Probe: what was the result?

1. **Acceptance of HIV Testing and Treatment Program**

- What information were you given about your pregnancy, being HIV+ and giving birth?
- What information would you have liked to have received from the health workers about HIV, treatment or how this might affect you or the baby?
- Who did you live with when you were tested for HIV?
- To what extent did you disclose your HIV status at that time?
  - *Probe: Did she experience difficulties disclosing her status to her sexual partner?*
  - *Probe: Did she experience difficulties disclosing her status to her relatives?*
  - *Probe: Did she experience difficulties disclosing her status to her friends?*
- Have you started treatment for HIV? How did you experience treatment initiation?

Probe: how long did it take to start treatment?

- Are you, or is your child receiving treatment despite stopping going to___________ *(name of facility from which she dropped-out)* for HIV care?
  - *If yes, probe: where are you receiving treatment?*
  - *If yes, probe: why do you prefer this facility to the former?*
  - *If no, probe: when did you stop receiving treatment?*
  - *If no, probe: why did you stop receiving treatment?*

- How are you feeling now?
- How is your child feeling now?

1. **EXPERIENCES with HIV Treatment Program**
2. **Health Facility Experiences**

- Describe your experience of going to the health facility to receive care for yourself or your children?
  - *Probe: distance challenges*
  - *Probe: financial challenges*
  - *Probe: busy schedule with household or farming matters*
  - *Probe: (subjective) feeling of good or poor health*
- How did you experience taking HIV treatment when you were pregnant or after you had given birth?
  - *Probe: food challenges*
  - *Probe: lack of spousal/family support*
  - *Probe: others (specify)*
- How did you experience providing treatment to your child?
- Did you experience any challenges **at the health facility** which affected the way you and your child were receiving HIV care? Please clarify
  - *Probe: any shortages of health commodities and staff*
  - *Probe: any problems with the way health services were delivered (staff attitude, waiting times, privacy etc)\*
- How well did the **health care providers** perform in providing care to you and your child to get care before you stopped going to the health facility? *Please clarify*
- How do/did you feel about someone coming to your house to encourage you to attend the clinic
- In what ways can the **health care providers** improve in delivery of health care to HIV-positive women and their children?

1. **Household Experiences**

- Who did you live with when you started receiving HIV care?
- What sort of support, if any, did you receive from your relatives?
  - *Probe: Did she receive psychological or financial support?*
- How well did **your spouse/family** perform in supporting you and your child to get care before you stopped going to the health facility? *Please clarify.*
- Did you experience any challenges **at home** which affected the way you and your child were receiving HIV care? Please clarify?
  - *Probe: any family discord?*
  - *Probe: any abuse from spouses and relations*
  - *Probe: any lack of home support*
  - *Probe: any lack of food*
  - *Probe: too busy with other household activities*
  - *Probe: others (specify)*
- In what ways can **spouses/families** improve in supporting HIV-positive women and their children to get HIV care at health facilities?

1. **Community Experiences**

- How well did **the community** (community leaders, religious leaders, local politicians, etc) perform in supporting you and your child to get care before you stopped going to the health facility? *Please clarify*
- Did you experience any challenges **in the community** which affected the way you and your child were receiving HIV care? Please clarify
  - *Probe: any stigma or discrimination*
  - *Probe: any negative rumors*
  - *Probe: any competing advise about HIV management (eg from healers or religious leaders)*
- In what ways can **the community** improve in supporting HIV-positive women and their children to get HIV care?

1. **Suggestions for improvement**

- Would you like to start accessing HIV care at the health facility again, why or why not?
- What would encourage/ help you to start accessing HIV care at the health facility again?
- What can be done to:
  - Improve uptake of HIV care services for HIV-positive mothers and their babies?
  - Encourage people to access HIV care at the health facility?
