# Supplementary material for: Factors associated with loss-to-follow-up of HIV-positive mothers and their infants enrolled in HIV care clinic: A qualitative study
Source: BMC Public Health. 2020 Mar 6;20:298. doi: 10.1186/s12889-020-8373-x (PMC7060526; doi:10.1186/s12889-020-8373-x)
Supplement: Supplementary file 2 — Additional file 2. HCW In-Depth Interview Guide. [file 12889_2020_8373_MOESM2_ESM.docx]

# Additional file 2 HCW In-Depth Interview Guide

**Instructions for Interviewer**

Your job as an interviewer is to facilitate honest and detailed responses about what the interviewee actually believes about any particular response to the questions below. This is not an exam for participants agreeing to be interviewed. There are no “right” or “wrong” answers. It is permissible to ask a participant to clarify a response if you do not understand. However, do not seek unnecessary clarification, causing the nature of the original response to change substantively. While the discussion should feel natural, avoid providing too much of your own personal insight, which may lead or sway a participant to reach your own pre-determined conclusion. Your job is to motivate the participant to expand on their own ideas, and allow them to reach conclusions on their own.

The questions below have been designed as to not solicit simple “yes” and “no” answers, but are open-ended in such a way that participants can answer them as they see fit, given their own experience and knowledge of the question. Your role as a guide is to keep participants from straying off topic. To accomplish this, you may ask for details, stories, anecdotes, descriptions of setting, opinions, attitudes, and perceptions about responses to answers that are already on topic. Avoid repeating a question, which, you feel, has already been adequately addressed. Thus, **it is not necessary to ask each and every question in the IDI guide in the sequence that has been provided**.

**Introduction for Participant**

You ………………………. *(the healthcare worker)* have been identified as a key stakeholder who can provide information and perspectives on mother-infant pair clinics, and maternal and paediatric HIV care at ………………………. *(name of facility)*. Please help us by answering the following questions based on your experiences, expertise and opinions learned through your work.

The questions are intended to help us identify reasons why some mothers and infants disengage or dropout from PMTCT care. The discussion will focus on the following factors:

- Personal or family factor
- Community Factors
- Health System factors

Please indicate whether you are willing to answer the questions below, as well as the extent to which you require your participation in this process to be confidential, by answering the following questions.

Are willing for your name and organization to be identified as a key informant in our final report?

YES  NO

Are willing for your opinions and perspectives to be accredited to you and your organization in our final report?

YES  NO

Do you understand that if you answered “NO” to both of the above questions, that we will keep all identifiable information about you anonymous?

YES  NO

**______________________________________________________________________________**

1. **Demographic Data**

Age :   years

Gender : ___________

Marital Status : ___________

Education Level : ___________

Occupation : ___________

For how long have you been working at this facility?  days/weeks/months/years *(tick applicable)*

______________________________________________________________________________

1. **Background**

- What sort of HIV care services do you provide to HIV-positive mothers and their children at this health facility?
- Over what period have you, personally, been providing the HIV services?
- On which days of the week do you provide HIV care services?
- On a typical clinic, how many HIV-positive mothers and their infants receive HIV care services?

1. **Women’s acceptance of HIV testing and treatment program**

- When women test positive for HIV, what information do you give them about their pregnancy and being HIV+?
- In general, to what extent do you think HIV-positive mothers understand the information that you provide them regarding HIV testing and the treatment?
- Are there specific areas that HIV-positive mothers appear not to understand well about their HIV care program?
- In general, to what extent do you think HIV-positive mothers and their infants adhere to the advice that you provide them?
- In general, do women find it easy/ difficult to start treatment? Why?

1. **EXPERIENCES with HIV Treatment Program**

- In your own view, what challenges do HIV-positive mothers face in accessing HIV care for themselves and their babies?
- In your own views, at what time point does most of the HIV positive mothers usually stop coming to the health facility to receive HIV care for themselves and their children?
- What are the main reasons why they stop accessing care for themselves and their children?

1. **Health Facility Experiences**

- To what extent does this **health facility and health care providers** contribute to the problem of mothers stopping accessing HIV care? Please explain?
  - *Probe: access to the facility*
  - *Probe: any shortages of health commodities and staff*
  - *Probe: any problems with the way health services were delivered (staff attitude, long waiting times, privacy, poor tracing etc)*
- How well are **health care providers** performing in providing support to HIV-positive mothers and their children to get care and to minimize the numbers who default from care? Please clarify.
- In what ways can the **health care providers** improve in delivery of health care to HIV-positive women and their children and prevent them from defaulting?

1. **Household Experiences**

- To what extent do the **family and/or spouses of HIV-positive mothers** contribute to the problem of mothers stopping accessing HIV care? Please explain?
  - *Probe: any family discord?*
  - *Probe: any disclosure problems?*
  - *Probe: any abuse from spouses and relations*
  - *Probe: any lack of home support*
  - *Probe: too busy with other household activities*
- How well are **spouses/families** performing in supporting HIV-positive mothers and their children to get HIV care and to minimize the numbers who default from care? Please clarify.
- In what ways can **spouses/families** improve in supporting HIV-positive women and their children to get HIV care at health facilities and prevent them from defaulting?

1. **Community Experiences**

- To what extent do **members of the general community** contribute to the problem of mothers stopping accessing HIV care?
  - *Probe: any stigma or discrimination*
  - *Probe: any negative rumors*
  - *Probe: any competing advise about HIV management (eg from healers or religious leaders)*
- How well is the **community** (community leaders, religious leaders, local politicians) performing in supporting HIV-positive mothers and their children to get care and to minimize the numbers who default from care? *Please clarify*.
- In what ways can **the community** improve in supporting HIV-positive women and their children to get HIV care and prevent them from defaulting? *Please clarify*

1. **Suggestions for improvement**

- What program could be put in place to minimize the numbers of HIV-positive mothers and their children who default from care? *Please clarify*
